# Clinicoepidemiology of Skin Diseases in Children Seen at the University Hospital Center Morafeno, Toamasina, Madagascar

**DOI:** 10.1155/2021/6456448

**Published:** 2021-09-09

**Authors:** Irina Mamisoa Ranaivo, Fandresena Arilala Sendrasoa, Malalaniaina Andrianarison, Moril Sata, Onivola Raharolahy, Dimby Stephane Ralandison, Lala Soavina Ramarozatovo, Fahafahantsoa Rapelanoro Rabenja

**Affiliations:** ^**1**^ Departments of Dermatology, University Hospital Morafeno, Toamasina, Madagascar; ^**2**^ Department of Dermatology, Joseph Raseta Befelatanana University Hospital, Antananarivo, Madagascar; ^**3**^ Department of Rheumatology, University Hospital of Morafeno, Toamasina, Madagascar

## Abstract

**Introduction:**

The child's skin diseases are common and very diverse. Many studies concerning pediatric dermatoses have been carried out in Africa and the rest of the world. Few epidemioclinical data reflect these skin diseases in children, especially in the east coast of Madagascar. We aim to describe the pattern skin diseases among children seen at the University Hospital Center Morafeno, Toamasina, Madagascar. *Patients and Methods*. A retrospective study over a 3-year period from January 2017 to December 2019 was conducted in children seen in the Dermatology Department of University Hospital Center Morafeno, Toamasina, with skin diseases.

**Results:**

During the study period, 347 children out of the 1584 new patients were retained with a sex ratio of 0.86. The mean age was 6.4 years old. The main diseases identified were skin infectious diseases (43, 23%), allergic dermatoses (24.21%), and autonomous dermatosis (15.56%).

**Conclusion:**

Our study revealed the importance of infectious and allergic dermatoses in Toamasina. It is necessary to carry out simple preventive actions such as hygiene.

## 1. Introduction

The child's skin diseases are common and very diverse. These skin disorders give uneasiness and anxiety of parents. Many studies concerning pediatric dermatoses have been carried out in Africa and the rest of the world [1–4]. Environmental factors play a significant role in skin disease. However, in Madagascar, particularly in Toamasina, this entity remains little studied. Toamasina is a city on the east coast of Madagascar with a hot and humid climate. We aim to describe the pattern skin diseases among children seen at the CHU Morafeno, Toamasina, Madagascar.

## 2. Patients and Methods

This is a retrospective study of all patients aged ≤15 years attending the outpatient dermatology who were examined at the Department of Dermatology at the University Hospital Center Morafeno in Toamasina, Madagascar, during 36 months (from January 2017 to December 2019). Epidemiologic data (age and gender) and diagnosis were collected from the patient's medical records. Patients with missing information were excluded from the study. The diagnoses reported on the medical records were based on clinical features and were confirmed by laboratory tests when indicated. The studied cases were further divided according to etiology, into the following groups: infectious skin diseases (bacterial skin infections, parasitic infestations, fungal infections, and viral infections), allergic dermatoses, autonomous dermatosis or inflammatory skin diseases, genodermatosis or congenital dermatosis, skin tumors, dysimmune diseases, and toxidermia (drug reaction). The data collections were carried out by the Excel 2010 software. The statistical analysis was processed on the EPI-INFO software version 7.2.2 16.

## 3. Results

During the study period, a total of 1584 new patients with skin diseases were seen, including 347 pediatric patients. Pediatric consultations represent 21.90% of all dermatology outpatient. Among the 347 pediatric patients, 159 (46.36%) were boys and 184 (53.64%) girls. There was a female preponderance (sex ratio: 0.86). Age ranges from newborn to 15 years (mean age 6.4years). The time between the onset of illness and the first consultation varied between one day to120 months with a mean delay of 12.18 months. The main diseases identified were skin infectious disease (43, 23%), allergic dermatoses (24, 21%), autonomous dermatosis (15, 56%), genodermatosis or congenital dermatosis (9, 51%), tumoral dermatosis (2.88%), dysimmune diseases (1.44%), and toxidermia (1.15%).  Table 1 shows the frequency and pattern of skin diseases groups according to the age ranges of the patients.

**Figure 1:**
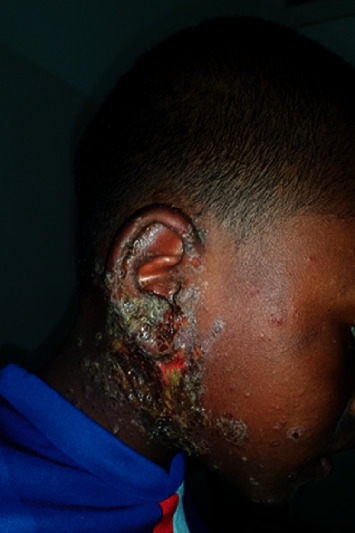
Impetigo.

Among the skin infectious disease, fungal infections were noted in 58 cases (31.67%) which consisted of pityriasis versicolor in 12.66% and tinea corporis in 10%. Regarding bacterial skin diseases, they were noted in 22 cases (14.67%) including impetigo (8%) (Figure 1), and there were 2 cases of skin tuberculosis. Viral infections represented 16.67% of infectious diseases, including molluscum contagiosum (10.66%). As for parasitic infestations, 41 cases were scabies (27.33%).  Table 2 provides the distribution of children according to infectious diseases.

**Figure 2:**
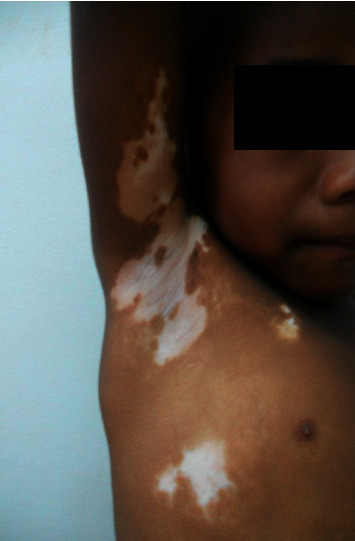
Vitiligo.

Allergic dermatoses were mainly represented by atopic dermatitis (72.62%). Most of the autonomous dermatoses were represented by vitiligo (48.15%) (Figure 2), followed by acne and psoriasis, respectively, 20% and 16.67%. Congenital dermatoses and/or genodermatoses mainly consisted of tuberous hemangiomas (42.42%), nevi (18.18%), and congenital ichthyosis (12.12%). These 3 groups of noninfectious dermatoses are given in Table 3. Skin tumors were mainly benign tumors (10 cases), of which 5 cases were keloids. There were 5 cases of autoimmune diseases including 2 cases of autoimmune bullous dermatosis and 3 cases of scleroderma. Finally, 4 cases of drug eruption were fixed pigmented erythema.

## 4. Discussion

The aim of this study was to describe the pattern skin diseases among children ≤15 years seen in Dermatological Outpatient Department at the University Hospital Center Morafeno, Toamasina. This study provided an overview of children's skin diseases in Toamasina. The present study found that 21.90% of the patients seen in dermatology in Toamasina were children; this frequency is comparable to the results of the literature [1, 2, 5]. The demographic profile of these children was superimposed on that of the literature. There was a predominance of the female gender in our study with 53.64%. Similar results were found in Côte d'Ivoire with a female predominance at 54.06% [3] and in Greece at 52.8% [6]. In Mali and India, it found a male predominance with, respectively, 55.10% and 58% [1, 4].

The average duration of disease progression before consultation is quite long, around 12.18 months. This could be explained by the recent opening of the Dermatology Department at the University Hospital Center Morafeno, Toamasina (2016). Poor economic and geographic accessibility to healthcare services by patients, recourse to traditional treatments, or self-medication delayed specialist consultations [7].

Infectious dermatoses was the largest group of skin disorders in childhood in our study constituting 43.23% of total 347 cases, followed by allergic dermatoses (24.21%) and autonomous dermatoses (15.56%). As in other African studies, infectious skin diseases were frequent and affected different age groups of children [1, 8]. The infectious diseases observed were fungal, parasitic, viral, and bacterial dermatoses. But the most common were superficial skin fungal infections (tinea corporis and pityriasis versicolor) and parasitic dermatoses in particular scabies. This frequency could be the consequence of poor hygiene and limited socioeconomic condition. The hot and humid climate also favours the appearance of infectious dermatoses [9].

After infectious dermatoses, there were allergic dermatoses, mainly atopic dermatitis as in most studies both in Africa and in Europe [1, 2]. These dermatoses affected all age groups. Lifestyle factors linked to urbanization were associated with an increased risk of allergic diseases in Africa [10, 11].

Concerning autonomous dermatoses, the most common were vitiligo, acne, and psoriasis. These dermatoses mainly affected the 11–15 age group. Although benign, they could have a negative impact on the quality of life of children, adolescents, and their families [12]. This high frequency for this age group is thought to be due to the aesthetic discomfort leading to a consultation. Vitiligo can appear soon after birth until late adulthood. But it appears before the age of 12 in 32–40% of cases [13]. And acne is often associated with adolescence but can appear at any age, especially in the prepubertal period [14].

Skin tumors were benign and appeared mostly in the 11–15-year age group. In fact, skin cancers are rare in children except in the case of preexisting dermatoses such as albino or xeroderma pigmentosum. These skin tumors were essentially keloids. The absence of comorbidities promotes good wound healing in children compared to adults due to good vascularisation of the skin. But hypertrophic and keloid scars are frequent in the prepubertal period as part of the hormonal peak [15, 16].  Table 4 provides some studies of skin diseases in children reported in the literature.

## 5. Conclusion

Childhood skin diseases are variable and frequent in dermatological outpatient. The epidemioclinical characteristics of dermatoses in children in Toamasina did not differ from skin disorders in children seen in Africa. Our study revealed the importance of dermatological conditions, in particular infectious and allergic, as well as autonomous dermatoses. It is necessary to carry out simple preventive actions such as hygiene to encourage and educate patients to come to the hospital in time for appropriate treatment.

## Figures and Tables

**Table 1:** The frequency and pattern of skin diseases groups according to the age ranges of the patients

Skin diseases	Age ranges					
	<1 year	1–5 years	6–10 years	11–15 years	Total	Percentage (%)
Infectious dermatoses	22	62	44	22	150	43.23
Autonomous dermatoses	01	15	14	24	54	15.56
Allergic dermatoses	11	30	25	18	84	24.21
Congenital dermatoses	15	08	06	04	33	9.51
Dysimmune dermatoses	0	0	02	03	05	1.44
Tumors skin	0	02	01	07	10	2.88
Toxidermia	0	03	01	0	04	1.15
Others	05	02	0	0	07	2.02
**Total**	**54**	**122**	**93**	**78**	**347**	**100**

**Table 2:** The distribution of children according to infectious diseases.

Dermatoses	Diagnosis	Number of patients (150)	Percentage (%)
Fungal infectious	Pityriasis versicolor	19	12.66
	Tinea corporis	15	10.00
	Tinea capitis	14	9.33
	Seborrheic dermatitis	10	6.66
	**Total**	**58**	**38.67**

Parasitic infestation	Scabies	41	27.33
	Cutaneous larva migrans	04	02.66
	**Total**	**45**	**30**

Viral infections	Molluscum contagiosum	16	10.66
	Gibert's pityriasis rosea	03	2.00
	Varicella	01	0.66
	Zona	02	1.33
	Herpes	01	0.66
	Warts	02	1.33
	**Total**	**25**	**16.67**

Bacterial skin diseases	Impetigo	12	08.00
	Furunculosis	07	0.46
	Abcess	01	0.66
	Cutaneous tuberculosis	02	1.33
	**Total**	**22**	**14.67**

**Table 3:** The distribution of children according to noninfectious skin diseases.

Dermatoses	Diagnostics	Number of patients	Percentage (%)
**Allergic dermatosis**	Atopic dermatitis	61	72.62
	Contact eczema	10	11.90
	Prurigo	12	14.29
	Urticaria	01	1.19
	**Total**	**84**	**100**

**Autonomous dermatoses**	Vitiligo	26	48.14
	Acne	11	20.37
	Psoriasis	09	16.67
	Lichen striatus	06	11.11
	Alopecia areata	02	3.07
	**Total**	**54**	**100**

**Congenital dermatoses**	Hemangioma	14	42.42
	Nevi	06	18.18
	Congenital ichthyosis	04	12.12
	Pityriasis rubra pilaris	03	09.09
	Neurofibromatosis	02	06.06
	Klippel–Trenaunay	01	03.03
	Bourneville tuberous sclerosis	01	03.03
	Anhidrotic ectodermal dysplasia	01	03.03
	Aplasia cutis congenita	01	03.03
	**Total**	**33**	**100**

**Table 4:** Comparative analysis of some studies of skin diseases in children reported in the literature.

Study	Country	Period study	Number of patients	Most frequent skin diseases
Ben Saif and Al Shehab [17]	Al-Khabar	January 2004 to January 2006	383	Dermatitis and eczema 30.30%
	Saudi Arabia			Infectious diseases 12.5%
				Pigmentary disorders 8.9%

Sacchidanand et al. [9]	Bangalore	January 2011 to June 2011	1 090	Infectious diseases 32.4%
	India			Eczema 20.66%
				Pigmentary disorders 7.4%

Vakirlis et al. [6]	Thessaloniki	January 2013 to December 2015	940	Dermatitis/eczema 31.4%
	Greece			Viral infection 12.5%
				Pigmentary disorders 7.3%

Özçelik et al. [18]	Erzincan	January 2014 to November 2016	10 115	Infectious diseases 24.62%
	Turkey			Eczema 21.95%
				Acne and follicular diseases 18.45%

Kourouma et al. [3]	Treichville	January 2010 to December 2014	3 587	Infectious diseases 29.2%
	Abidjan			Immunoallergic dermatitis 29.1%
				Inflammatory dermatoses 26.67%

Kiprono et al. [19]	Tanzania	September 2012 to August 2013	340	Infectious diseases 43.5%
				Eczema dermatitis 28.5%
				Pigmentary disorders 7.4%

Miotto et al. [20]	Sao Paolo	January 2017 to December 2017	2 330	Atopic dermatitis 18.3%
	Brazil			Genodermatoses 14.2%
				Infectious diseases 12.6%

Present survey	Toamasina	January 2017 to December 2019	347	Infectious diseases 43.23%
	Madagascar			Allergic dermatoses 24.21%
				Autonomous or inflammatory dermatoses 15.56%

## Data Availability

The data used to support the findings of this study are included within the article.
